# Supplementary material for: Selective pressures of platinum compounds shape the evolution of therapy-related myeloid neoplasms
Source: Nat Commun. 2024 Jul 17;15:6025. doi: 10.1038/s41467-024-50384-z (PMC11255340; doi:10.1038/s41467-024-50384-z)
Supplement: Supplementary file 1 — Supplementary Information [file 41467_2024_50384_MOESM1_ESM.pdf]

Supplementary information

Selective pressures of platinum compounds shape the evolution of therapy-related myeloid neoplasms.

Eline J.M. Bertrums*[1,2,3], Jurrian K. de Kanter*[1,2], Lucca L.M. Derks[1,2], Mark Verheul[1,2], Laurianne Trabut[1,2], Markus J. van Roosmalen[1,2], Henrik Hasle[4], Evangelia Antoniou[5,6], Dirk Reinhardt[5,6], Michael N. Dworzak[7,8], Nora Mühlegger[7], Marry M. van den Heuvel-Eibrink[1,9], C. Michel Zwaan[1,3], Bianca F. Goemans[1], Ruben van Boxtel[1,2]

Affiliations
1. Princess Máxima Centrum for pediatric oncology, Utrecht, the Netherlands
2. Oncode Institute, Utrecht, the Netherlands
3. Department of Pediatric Oncology/Hematology, Erasmus Medical Center – Sophia Children's Hospital, Rotterdam, the Netherlands
4. Department of Pediatrics, Aarhus University Hospital, Aarhus, Denmark
5. Clinic of Pediatrics III, University Hospital of Essen, Essen, Germany
6. AML-BFM Study Group, Germany
7. St. Anna Children's Cancer Research Institute, Vienna, Austria
8. St. Anna Children's Hospital, Department of Pediatrics and Adolescent Medicine, Medical University of Vienna, Vienna, Austria
9. Utrecht University, Utrecht, the Netherlands

* These authors contributed equally

Corresponding author: Ruben van Boxtel, R.vanBoxtel@prinsesmaximacentrum.nl

Supplementary Figures

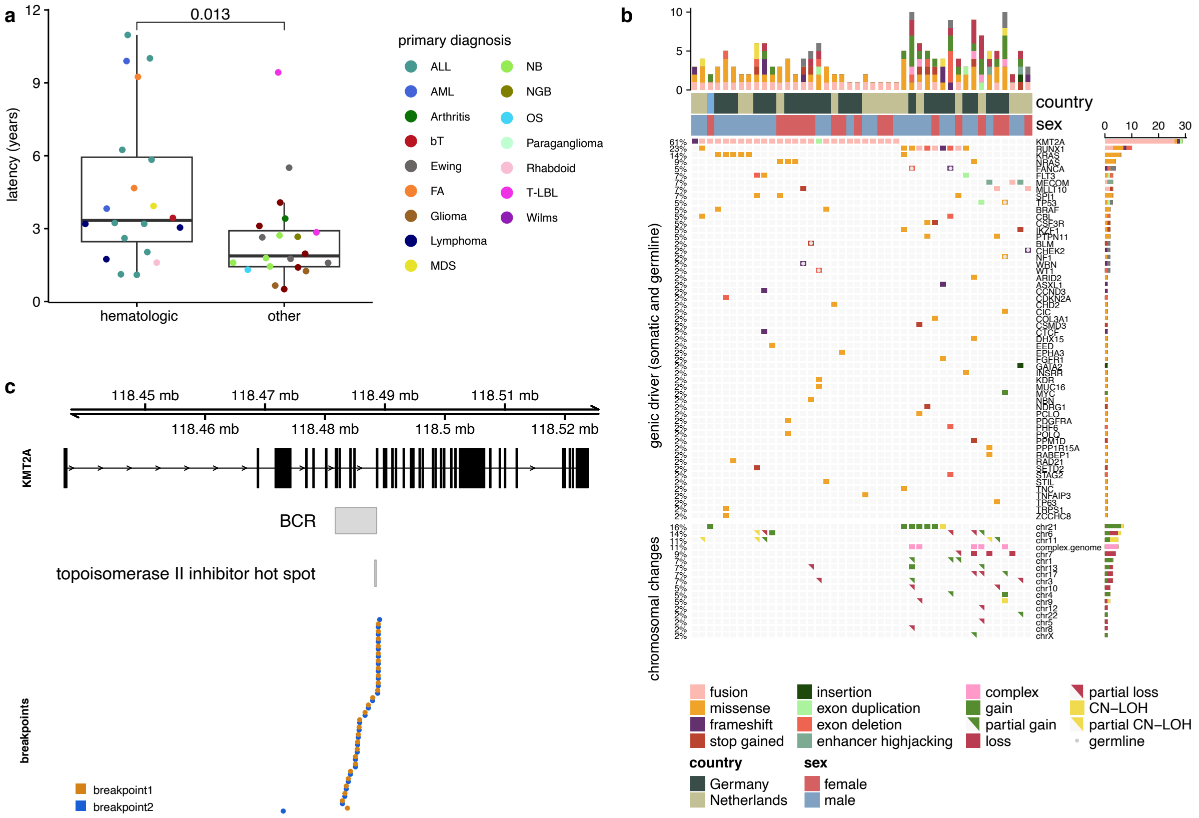

Supplementary Figure 1. Additional cohort details

a) Boxplot depicting the latency time in years between the first diagnosis and t-MN development. Colors represent first diagnosis. ALL: acute lymphoblastic leukemia; AML: acute myeloid leukemia; bT: beta-thalassemia; FA: Fanconi anemia; MDS: myelodysplastic syndrome; NB: neuroblastoma; NGB: neuroganglioblastoma; OS: osteosarcoma; SCT: allogenic stem cell transplantation; TLBL: T-cell lymphoblastic lymphoma. Two-sided Wilcox-test. The box plots depict the median (center line), 25th and 75th percentiles (box), and the largest values, no more than 1.5* the interquartile range (whiskers). **b)** Oncoprint with all identified driver mutations in all t-MN. In contrast to main Fig. 1e also mutations found in single t-MN were included. The bar plots on top represent the number of driving events present in each sample. The bar plots on the right represent the number of patients with the driver. CN-LOH: copy neutral loss of heterozygosity. **c)** *KMT2A* (MLL) breakpoints from t-MN patients in our cohort. Indicated on top are the general *KMT2A* breakpoint cluster region (BCR) and the hot spot associated with topoisomerase II inhibitors (TOP2i). *Source data are provided as a Source Data file.*

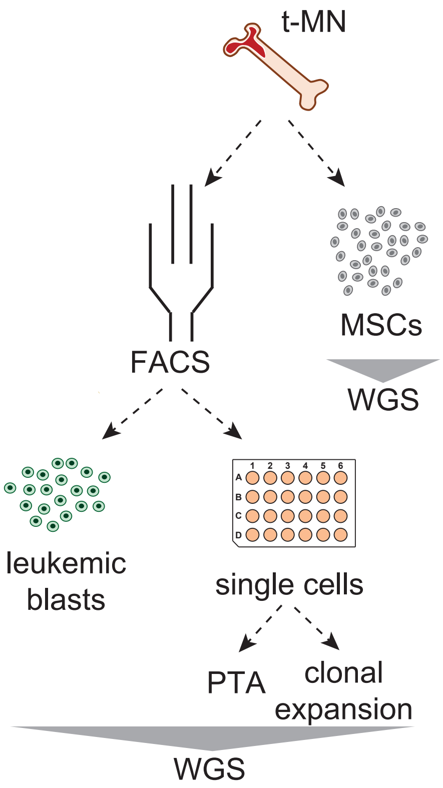

<u>Supplementary Figure 2. Experimental setup</u>

A schematic overview of the experimental setup of this study. In short, bone marrow biopsies at time of t-MN were collected. Blasts and HSPCs were purified by FACS. Blasts were sorted in bulk and single cell in a 96-wells plate for primary template-directed amplification (PTA). Single HSPCs were sorted in a 384-wells plate for clonal expansion and in a 96-wells plate for PTA. Mesenchymal stromal cells (MSCs) were plated and expanded *in vitro.*

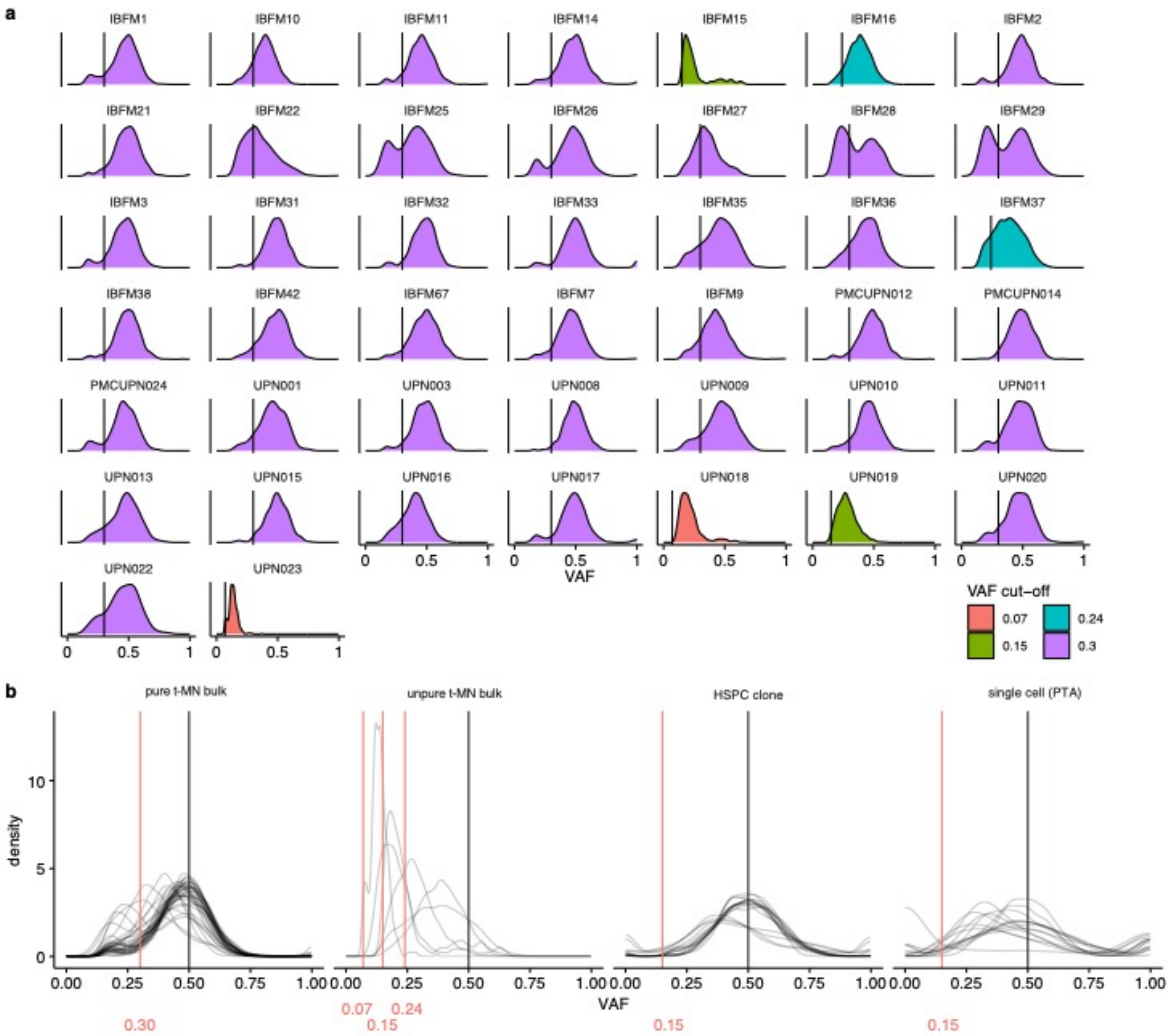

Supplementary Figure 3. VAF distributions and cut-offs

a) The VAF distributions of the different types of samples included in the manuscript. The blue numbers indicate the VAF cut-offs used for each type. **b)** The VAF distributions of the different bulk t-MN samples and the VAF cut-off used. *Source data are provided as a Source Data file.*

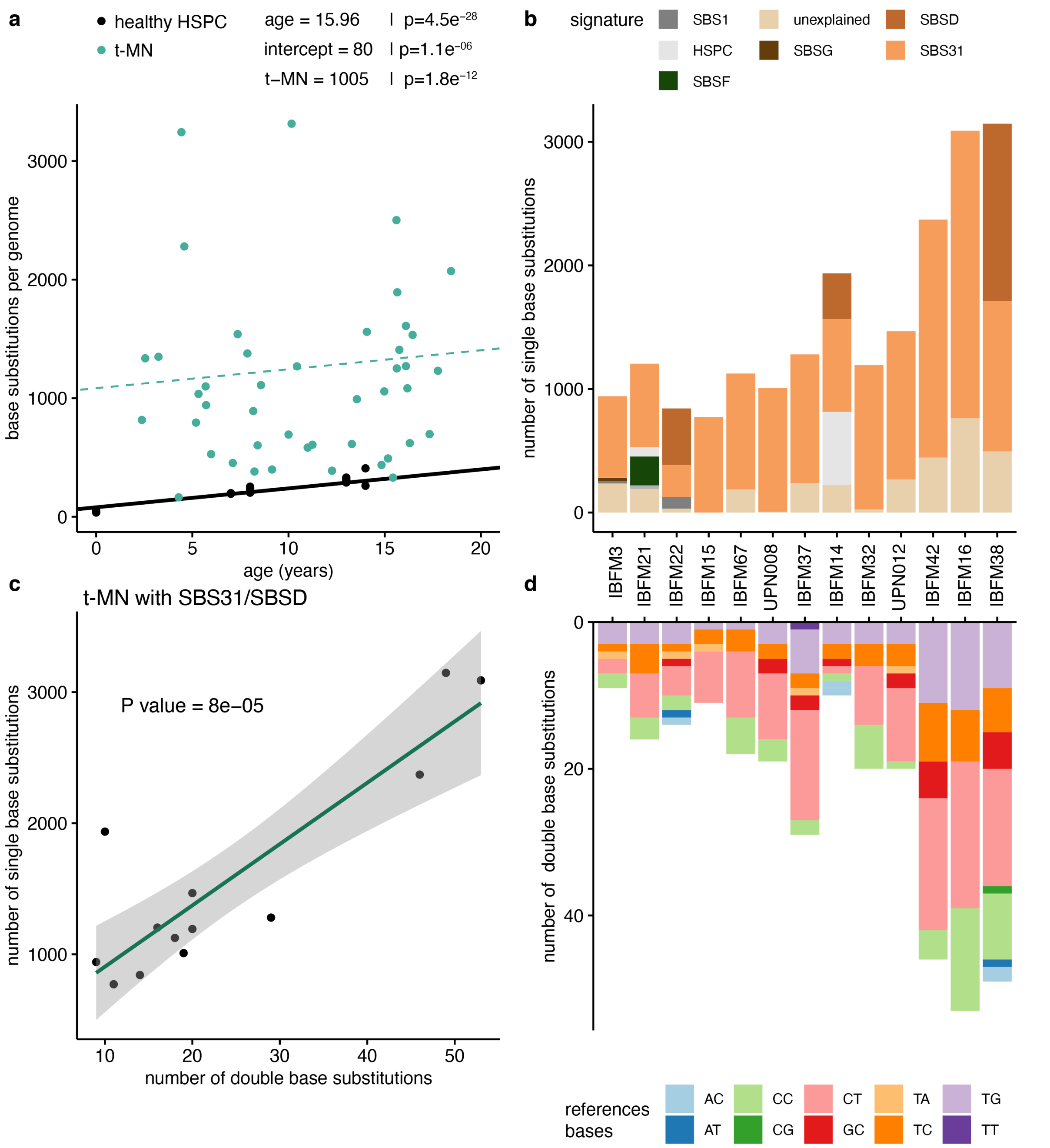

Supplementary Figure 4. Mutation burden of patient with platinum-related mutations

a) Mutation accumulation of t-MN (colored dots) compared to the baseline of healthy hematopoietic stem and progenitor cells (HSPCs; black dots). A linear mixed-effects model was run on both the baseline and t-MN data, taking into account the donor, the age and the mutation load. The effects for age (per year), the intercept of the baseline with the y-axis (number of mutations at birth), and the additional effect for t-MN are stated, together with p-values. t-MN on average had 1005 additional mutations compared to the healthy baseline with a p-value of $3.3*10^{-12}$. Conditional R^2=0.998 **b)** The signature contribution of t-MN that harbored a contribution of the platinum-related signatures SBS31 and/or SBSD. **c)** A linear model of the single base and double base substitutions in the t-MN patients depicted in (b) where n_snv = 461 + 46*n_dbs, adjusted R^2=0.74. **d)** The number and type of double base substitutions of the same t-MN depicted in (b). These are similar to DBS5, the COSMIC double base substitutions signature linked to platinum-based compound exposure. *Source data are provided as a Source Data file.*

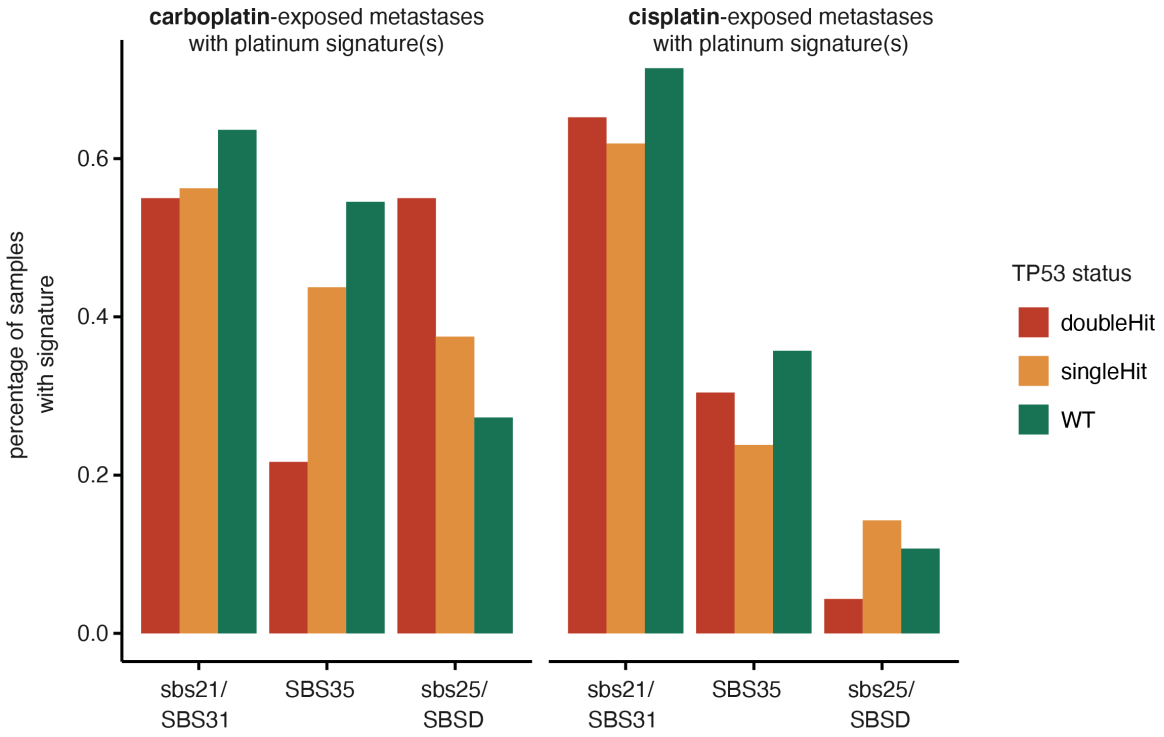

<u>Supplementary Figure 5: Platinum-related signatures in metastases by *TP53* status.</u> Carboplatin and cisplatin exposed metastases from a previously described cohort by Priestley *et al.*[36] that displayed any cisplatin-related signatures (SBSD/sbs25, SBS31/sbs21, SBS35) after bootstrapped refitting (n=100) were included. The bars represent the percentage of samples that had contribution of each signature. The samples were split by *TP53* status: wild-type (WT), single hit, double hit (most often a somatic mutation and a loss of the wild-type allele). *Source data are provided as a Source Data file.*

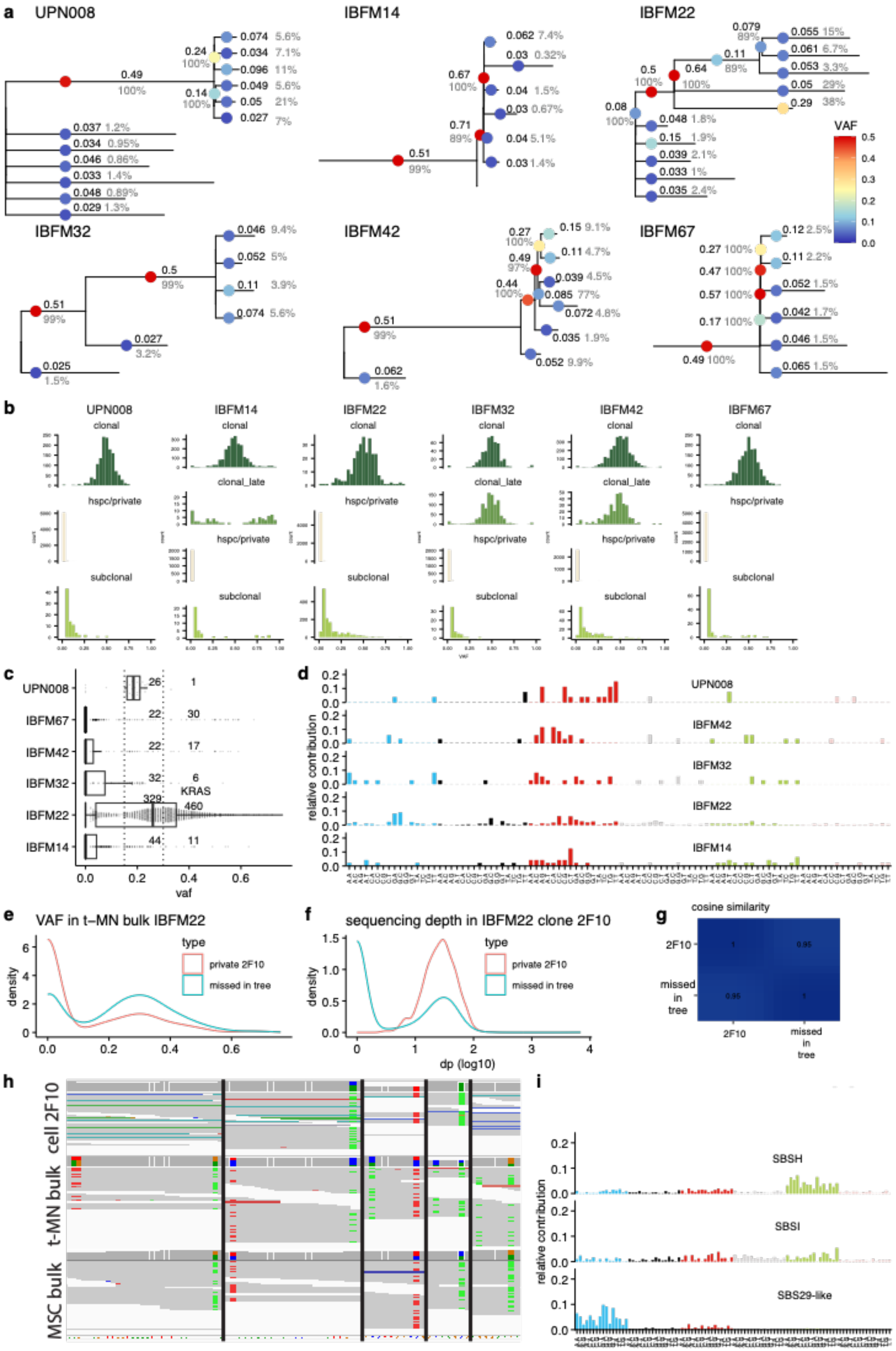

Supplementary Figure 6. Validation of the phylogenetic trees using WGS of bulk t-MN.

a) The t-MN trees shown in Figure 3 and 4. The number below each branch indicates the percentage of the mutations in the branch that is found in any read in the bulk t-MN WGS data. The number above the branch indicates the average variant allele frequency (VAF) of the mutations that are at least supported by one read in the bulk t-MN WGS data. The color of the dot corresponds with this value. **b)** The t-MN bulk VAF distribution of the mutations in the trees in Figure 3 and 4, grouped by category. **c)** The t-MN bulk VAF of the mutations not found in the phylogenetic trees in Figure 3 and 4. The numbers indicate the number of mutations between 0.15 and 0.3 and above 0.3. "*KRAS*" indicates a *KRAS* p.G12V mutation in IBFM32. The box plots depict the median (center line), 25th and 75th percentiles (box), and the largest values, no more than 1.5* the interquartile range (whiskers). **d)** The mutational profiles of the mutations with a VAF higher than 0.15 from (a). **e)** The IBFM22 bulk t-MN VAF of mutations not identified in the corresponding tree (similar to (a)) and of the mutations found in the subclonal/private or cell 2F10. **f)** The sequencing depth in cell 2F10 of the same mutation sets as in (c). **g)** The cosine similarity of the 96-trinucleotide mutational profiles of the two sets of mutations shown in (c) and (d). **h)** Examples of mutations that are found in bulk t-MN, but not present in any cell of the tree ("non-tree" in (c) and (d)) and have reads in cell 2F10 that cover the mutation site, but for which no reads are present in 2F10 that support the mutation (the blue peak between 1 and 2 in panel f). Germline mutations in close proximity indicate that in most cases only one of the two alleles was amplified in 2F10, in most cases the reference allele of the somatic mutation site. **i)** The 96-trinucleotide mutational profiles of the mutational signatures identified in the phylogenetic trees, SBSH and SBSI. *Source data are provided as a Source Data file.*

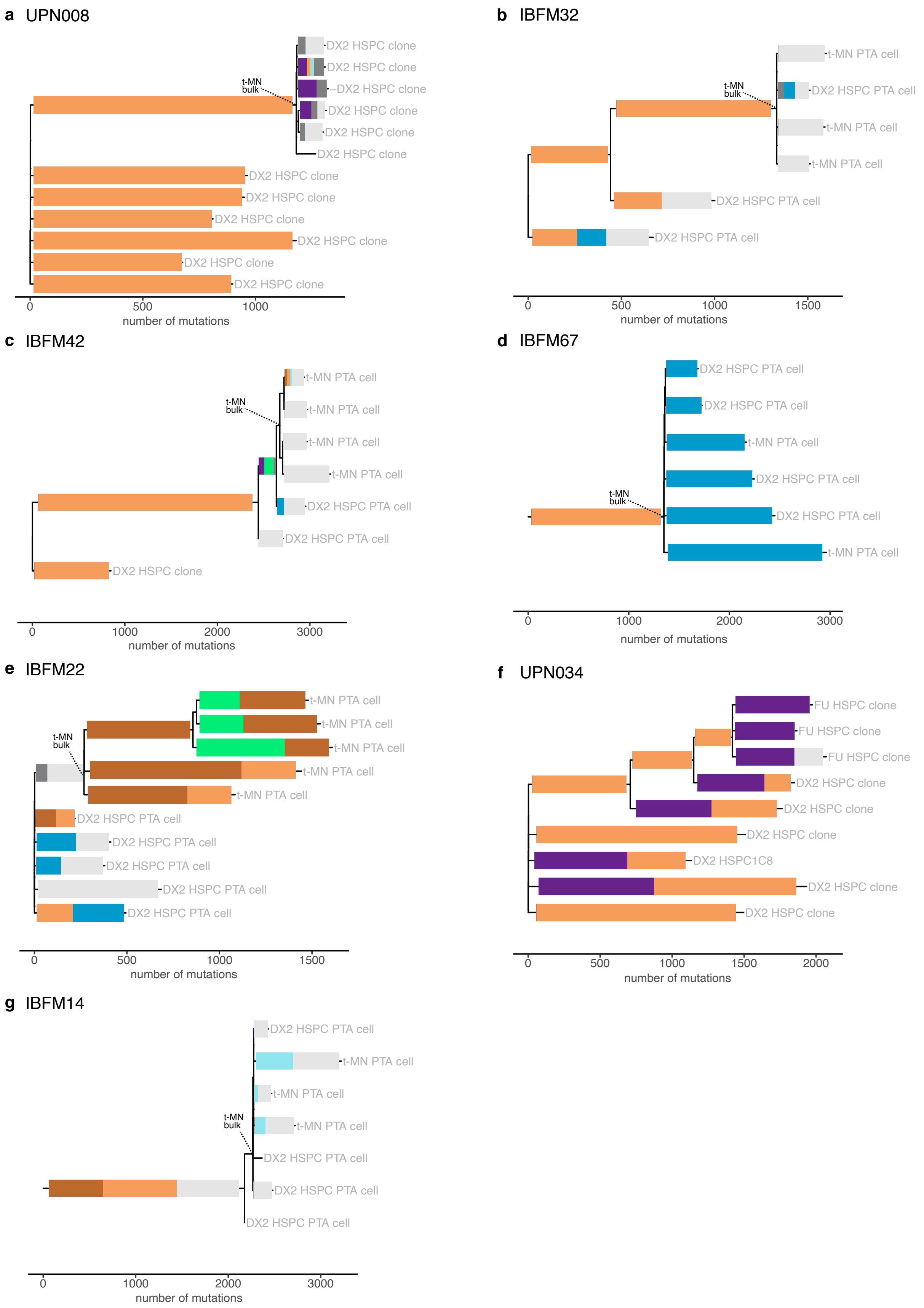

<u>Supplementary Figure 7. The phylogenetic trees in an alternative style.</u>

The phylogenetic trees depicted in Figure 3 and 4, but with the signature contributions per branch in bar plots in the tree. The colors of the bar plots in the branches depict the

mutational signatures that contribute to the mutations. Per signature, the same color is used as in the figures of the main manuscript. In addition, the type of cell/clone is indicated in words instead of symbols. Finally, the information of the drivers is removed. **a)** Phylogenetic tree of clonally expanded hematopoietic stem and progenitor cells (HSPCs) and bulk t-MN blasts of patient UPN008. **b)** Similar to (a), but for patient IBFM32. Bulk t-MN blasts and single cells were sequenced. Cells that are indicated as “PTA” are single-cell sequenced using primary template-directed amplification. **c)** Similar to (a), but for patient IBFM42. Besides the bulk t-MN blasts, single-cell t-MN blasts, single HSPCs and an HSPC clone were sequenced. **d)** Similar to (a), but for patient IBFM67. Bulk t-MN blasts, single t-MN blasts and single HSPCs were sequenced. **e)** Similar to (a), but for patient IBFM22. Bulk t-MN blasts, single t-MN blasts and single HSPCs were sequenced. **f)** Similar to (a), but for patient UPN034. Clonally expanded HSPCs were sequenced, bulk blasts were unavailable. **g)** Similar to (a), but for patient IBFM14. Bulk t-MN blasts, single t-MN blasts and single HSPCs were sequenced. *Source data are provided as a Source Data file*.

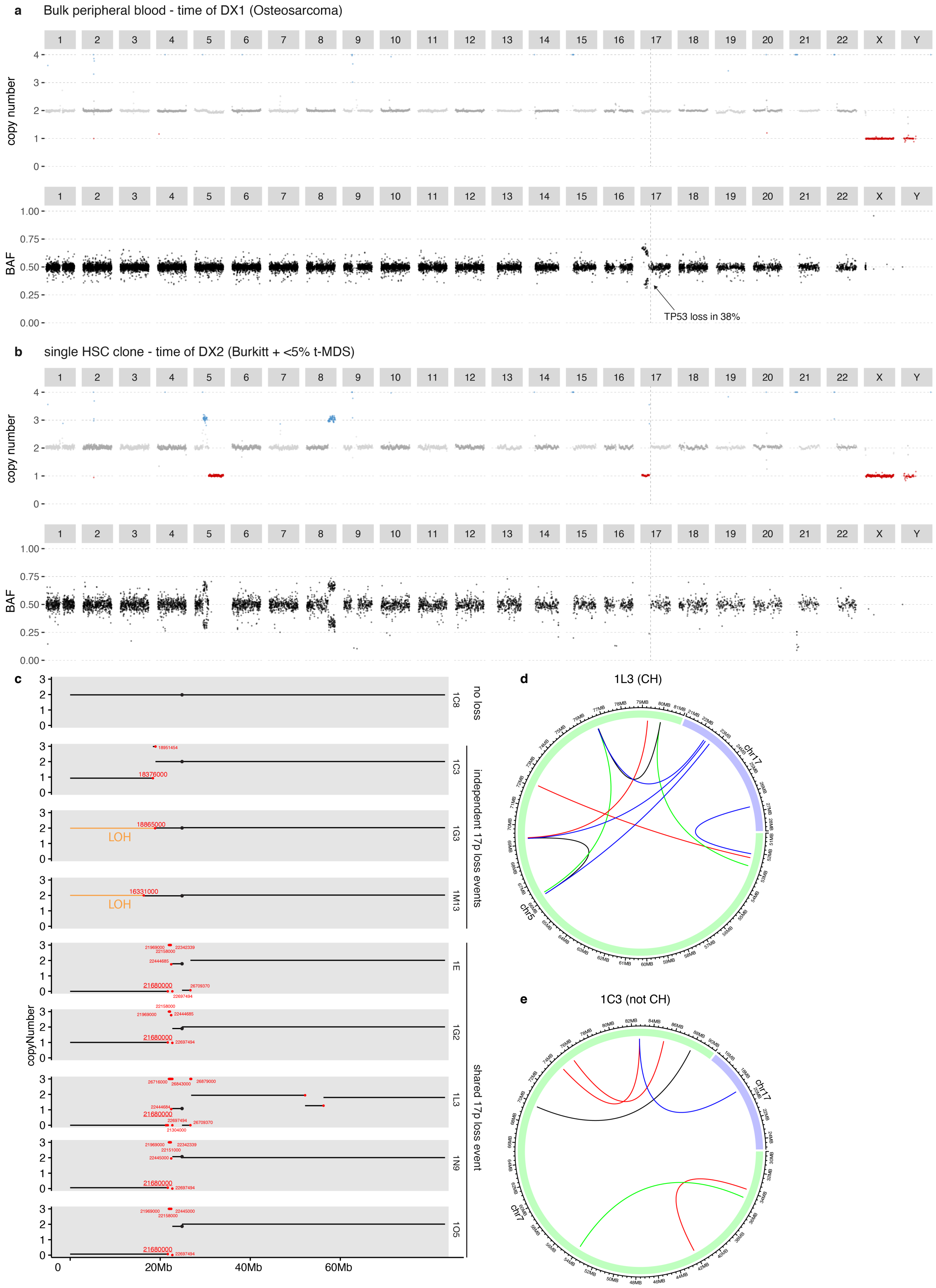

Supplementary Figure 8. Details of the UPN034 17p loss.

a) Copy number plot (top) and B allele frequency (BAF) plot (bottom) of whole genome sequencing (WGS) data of peripheral blood of patient UPN034 at the time of osteosarcoma (DX1). Data from the diagnostics department of our institute. **b)** Similar to (a) but for a bone marrow HSPC at time of DX2. **c)** A detailed list of breakpoints and copy number regions

identified in the cells of patient UPN034. **d)** The structural variants in clonal hematopoiesis (CH) clone 1L3 between chromosome 5 and 17, which led to the 17q loss in 5 cells (shown in c). **e)** Similar to (d), but for the events between chr17 and chr7 of non-CH clone 1C3*. Source data are provided as a Source Data file*

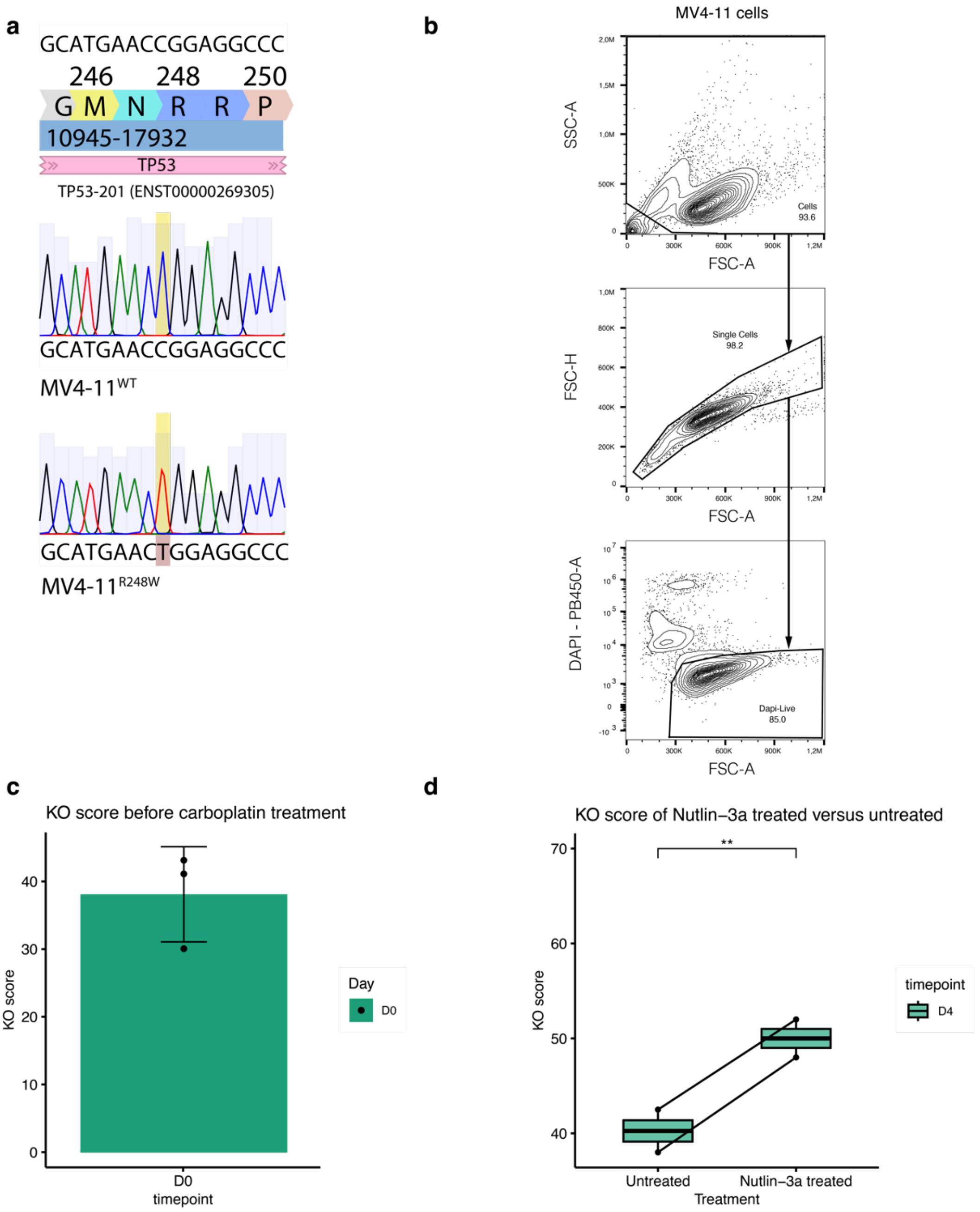

<u>Supplementary Figure 9. Assessment of knock-out status and isolation of *in vitro TP53*-deficient models</u>

a) Representative genotyping results of MV4-11^{wt} (top), MV4-11^{R248W} (bottom) lines aligned to ENST00000269305.9. The nucleotide variant leading to R248W is highlighted in yellow, the variant leading to N246T in blue. **b)** Representative gating of 1) cells (top), single cells (middle), and DAPI-negative cells (bottom, Dapi-Live) for MV4-11 cells. **c)** The KO score of the TP53 KO conditions based on ICE analysis (Synthego) at the start of carboplatin treatment (day 0). Error bars represent the standard deviation of the mean and each data point represents a biological replicate (n = 3 independent experiments). **d)** Validation of loss of p53 function due to CRISPR-Cas9 targeting. The KO score of the TP53 KO conditions based on ICE analysis (Synthego) with and without nutlin-3a treatment. The KO score was compared for n = 2 independent experiments with unique biological donors using a paired T-test (p = 0.0082). ** = p<0.01. *Source data are provided as a Source Data file.*

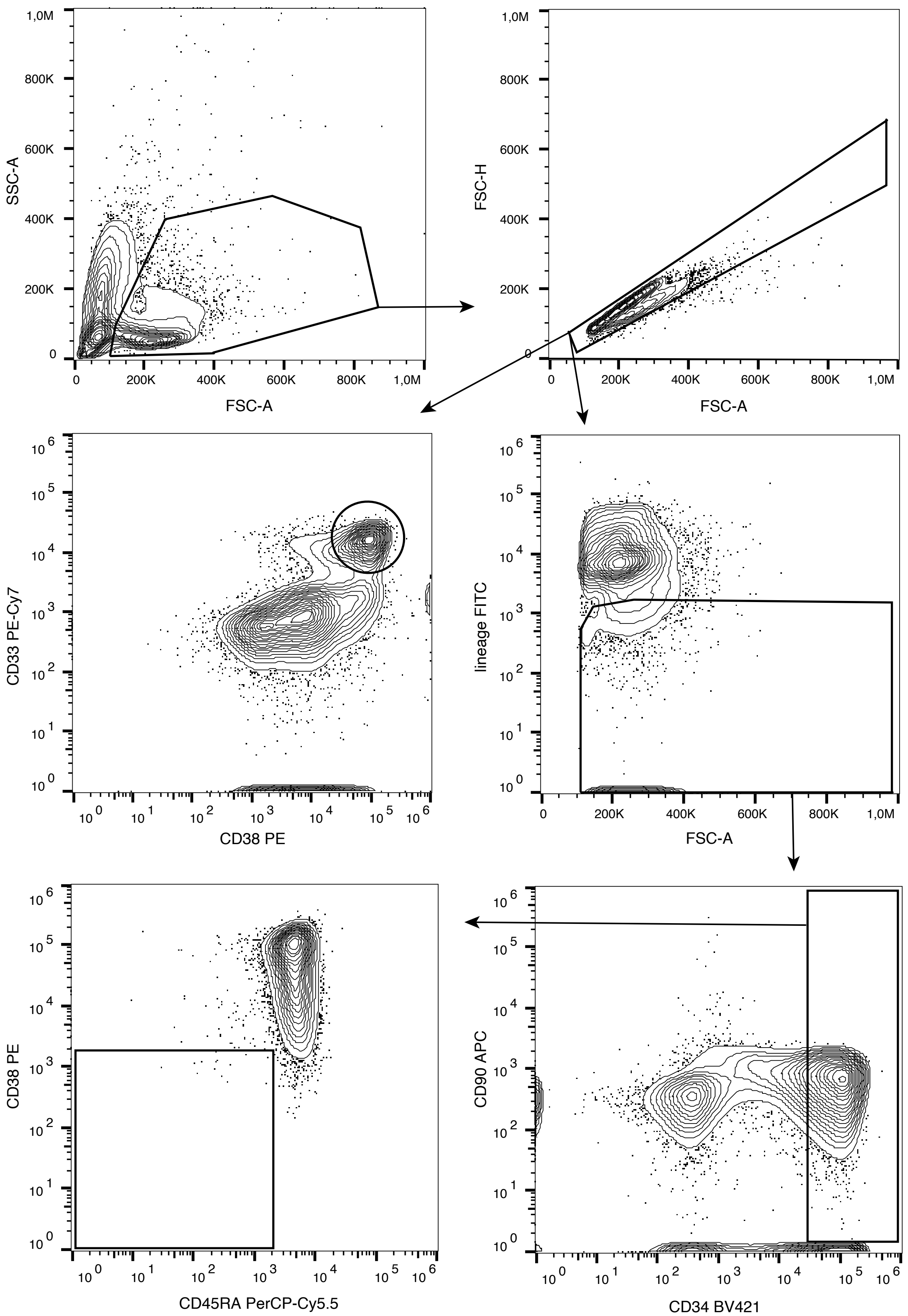

Supplementary Figure 10. Flow cytometry sorting strategy of blasts and hematopoietic stem and progenitor cells (HSPCs)

Representative fluorescence activated cell sorting (FACS) plot for purification of AML blasts (in IBFM t-AML sample these were CD33+CD38+, see middle left panel) and single cell HSPCs (bottem left panel: lin-CD34+CD38-CD45RA-).

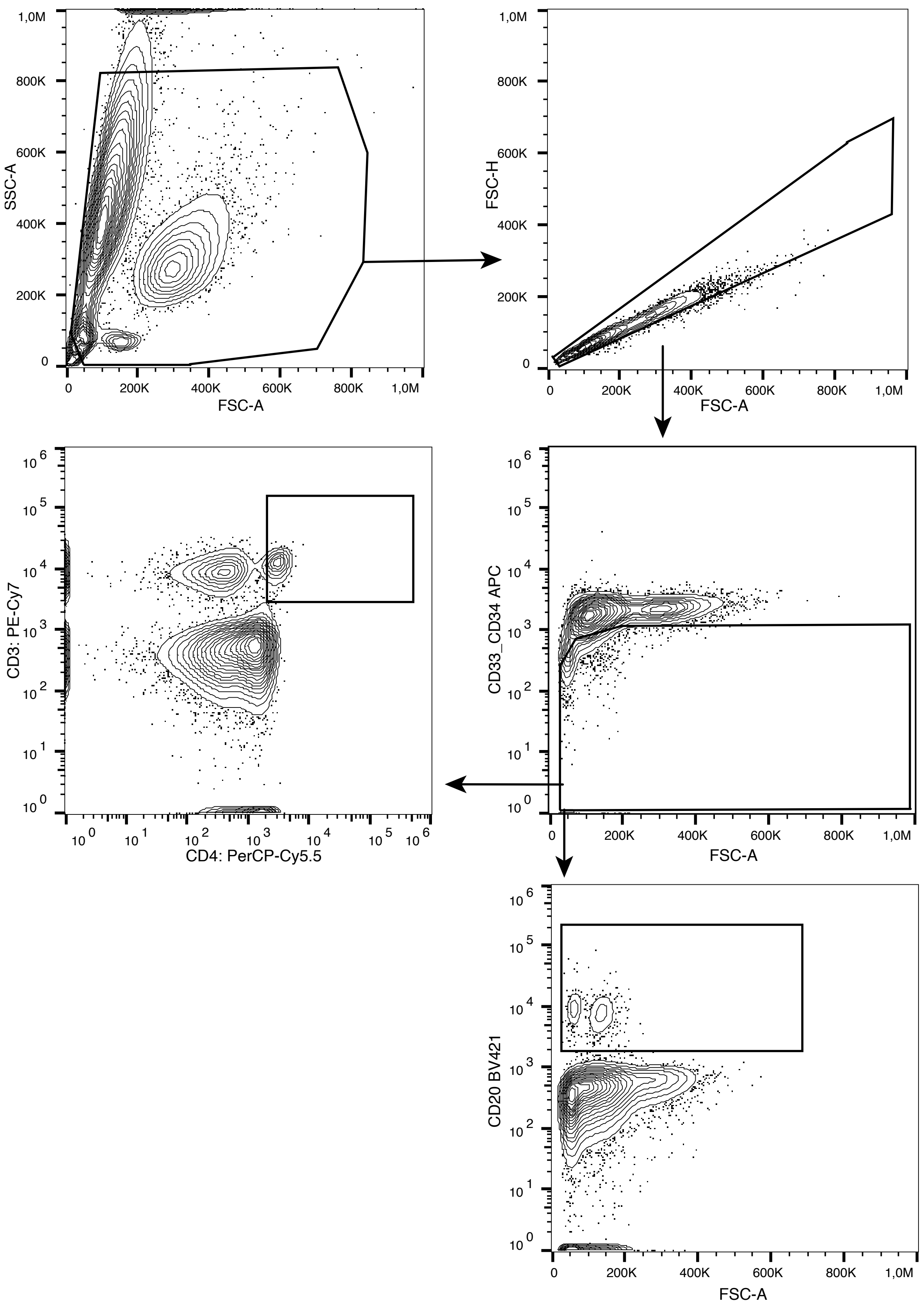

<u>Supplementary Figure 11. Flow cytometry sorting strategy of B-cells and T-cells</u>
Representative fluorescence activated cell sorting (FACS) plot for purification of B-cells (CD33-CD34-CD and CD4+ T-cells. Data shown is from IBFM21.

Supplementary Tables

Supplementary Table 1. Clinical patient and treatment information.

Patient ID	Gender	Primary cancer / *treatment protocol*	Age Dx1 (y)	Chemotherapy agents (extracted from protocol*)	Allo SCT	RT	Age t-MN (y)
IBFM01	M	ALL<br>*ALL-BFM-2000*	2.3	Vincristine, Daunorubicin, Asparaginase, MTX, Cyclophosphamide, 6-MP, ARA-C, Doxorubicin, 6-TG<br>If HR: + Ifosfamide, Vindesine	NA	NA	8.6
IBFM02	M	ALL<br>*ALL-BFM-2000*	3.7	Vincristine, Daunorubicin, Asparaginase, MTX, Cyclophosphamide, 6-MP, ARA-C, Doxorubicin, 6-TG<br>If HR: + Ifosfamide, Vindesine	No	No	5.7
IBFM03	F	Osteosarcoma<br>*NA*	11.6	Etoposide and from the alkaloid and/or taxane class	No	No	13.6
IBFM07	F	ALL<br>*ALL-BFM-92*	5.2		Yes	NA	16.2
IBFM09	F	ALL<br>*AIEOP ALL BFM*	3.5	Asparaginase, MTX, Daunorubicin, Docorubicin, ARA-C, Cyclohosphamide, 6-MP<br>Potentially: Etoposide, Ifosfamide, Vindesine	No	No	4.6
IBFM10	M	Ewing Sarcoma<br>*Euro-Ewing 99*	14.5	Vincristine, Ifosfamide, Doxorubicin, Etoposide, Actinomycin D<br>If R1: potentially + Cyclophosphamide<br>If R2: potentially + Busulfan, Melphalan<br>If R3: potentially + Melphalan or Treosulfan/Melphalan or Busulphan/Melphalan	NA	NA	16.1
IBFM11	M	MDS<br>*NA*	12.4	From the alkylating class and drugs from the alkaloid and/or taxane class	No	No	16.3
IBFM14	M	Ewing Sarcoma<br>*Euro-Ewing 99*	12.9	Vincristine, Ifosfamide, Doxorubicin, Etoposide, Actinomycin D<br>If R1: potentially + Cyclophosphamide<br>If R2: potentially + Busulfan, Melphalan<br>If R3: potentially + Melphalan or Treosulfan/Melphalan or Busulphan/Melphalan	NA	NA	18.4
IBFM15	F	Neuroblastoma (St. IV)<br>Studytherapy NB 2004 (HR) + 2x N8	3.7	Cisplatin, Etoposide, Vindesin, Vincristine, Dacrabaxine, Ifosfamide, Doxorubicin	Yes	NA	5.2
IBFM16	F	Neuroblastoma (St. III)<br>*NB2004*	2.8	Cisplatin, Etoposide, Vindesin, Vincristine, Dacarbazine, Ifosfamide, Doxorubicin, 13-cis-retinoic acid<br>If MR: + Cyclophosphamide	No[1]	Yes[1]	4.4
IBFM21	M	Paraganglioma<br>*GPOH MET 97*		Vincristine, Ifosfamide, Doxorubicin, Carboplatin, Etoposide	No[1]	No	10.4
IBFM22	F	Opticus glioma<br>*SIOP-LGG-2004 (NF1)*	7.5	Vincristine, Carboplatin (if allergic Cisplatin and Cyclophosphamide)	No[1]	NA	8.2
IBFM25	M	Nephroblastoma<br>*SIOP-2001*	5.4	Actinomycin D, Vincristine<br>Potentially: + Doxorubicin, Etoposide, Carboplatin, Cyclophosphamide	No[1]	NA	8.2
IBFM26	M	Fanconi anemia<br>*NA*	± 10	NA (pre-treatment t-MN with hydroxyurea 2 weeks before)	NA	NA	14.8
IBFM27	M	Hodgkin lymphoma<br>*EURO NET PHL*	5.3	Vincristine, Doxorubicin, Etoposide, Dacarbazine, Cyclophosphamide	NA	NA	8.4
IBFM28	M	Atypical rhabdoid tumor<br>*NA*	NA	Doxorubicin, MTX, Ifosfamide, Carboplatin, Etoposide, Vincristine, Cyclophosphamide, Actinomycin D, Thiotepa	NA	Yes[2]	2.5
IBFM29	F	systemic JIA, HLH[3]<br>*EURO HIT HLH*	0.9	ATG, Dexamethasone, Etoposide, MTX	No	No	4.3
IBFM31	M	c-ALL<br>*AIEOP BFM-ALL 2009 HR*	13.3	Vincristine, Daunorubicin, MTX, Aparaginase, Cyclophosphamide, ARA-C, 6-MP, Vindesine, Ifosfamide, Etoposide, Doxorubicin, 6-TG<br>Potentially: + Fludarabine, Daunoxome	No	No	16.4
IBFM32	M	(Osteosarcoma)<br>Pre-B-ALL<br>*AIEOP BFM-ALL 2009 HR*	15.1	*(Osteosarcoma treatment: NA)*<br>Vincristine, Daunorubicin, MTX, Aparaginase, Cyclophosphamide, ARA-C, 6-MP, Vindesine, Ifosfamide, Etoposide, Doxorubicin, 6-TG<br>Potentially: + Fludarabin, Daunoxome	No	No	15.6
IBFM33	M	Intrathoracic Ewing sarcoma<br>*Ewing 2008*	13.5	Vincristine, Ifosfamide, Doxorubicin, Etoposide, Actinomycin D<br>If R1: potentially + Cyclophosphamide<br>If R2: potentially + Busulfan, Melphalan<br>If R3: + Cyclophosphamide, potentially + Treosulfan/Melphalan	No	Yes	16.1
IBFM35	M	Fanconi Anemia<br>*NA*	± 9	Oxymetholon	Yes	NA	17.3
IBFM36	F	Pre-B-ALL<br>*AIEOP BFM-ALL 2009*	NA	Vincristine, Daunorubicin, MTX, Asparaginase, Cyclophosphamide, ARA-C, 6-MP, Leukovorin, Doxorubicin, 6-TG	No	NA	2.4

				If HR: + Vindesine, Ifosfamide, Etoposide, Doxorubicin, potentially + Fludarabine/Daunoxome			
IBFM37	F	Neuroblastoma *NB 2017 (HR)*	0.5	Cisplatin, Etoposide, Vindesine, Vincristine, Dacarbazine, Ifosfamide, Doxorubicin	No	NA	3.2
IBFM38	M	Nephrobastoma *SIOP-2001*	0.7	Actinomycin D, Vincristine<br>Potentially: + Doxorubicin, Etoposide, Carboplatin, Cyclophosphamide	No	No	10.2
IBFM42	M	Osteosarcoma *EURAMOS-1/COSS*	12.5	Doxorubicin, Cisplatin, MTX<br>Potenitally (if HR): Ifosfamide, Etoposide	N0	No	15.6
IBFM43	M	ALL *AIEOP BFM-ALL 2009*	4.2	Vincristine, Daunorubicin, MTX, Asparaginase, Cyclophosphamide, ARA-C, 6-MP, Leukovorin, Doxorubicin, 6-TG<br>If HR: + Vindesine, Ifosfamide, Etoposide, Doxorubicin, potentially.+ Fludarabine/Daunoxome	No	No	5.9
IBFM67	F	Osteosarcoma + relapse *EURAMOS-1*	13.7	Doxorubicin, Cisplatin, MTX	No	No	17.8
UPN001	F	Burkitt *LMB 2001*	11.5	Cyclophosphamide, ARA-C, MTX, Doxorubicin, Vincristine, Etoposide,	No	No	13.2
UPN002	F	B-ALL	3.9	Vincristine, Daunorubicin, MTX, ARA-C, Asparaginase	No	No	5.0
UPN003	M	ALL *ALL10, ALL-R3, ALL 11 HR + ADHOC*	5.7	At time of FU: MTX, Vincristine, ARA-C, PEG-asparaginase, Daunorubicin, Cyclophosphamide, 6-MP, Doxorubicin, Mitoxantrone, 6-TG<br>After FU new: ATG, BuFluClo, Teniposide, allogenic MUD-SCT	Yes, 2x	NA	15.7
UPN004	M	B-ALL	15.5	Vincristine, Daunorubicin, MTX, ARA-C, Asparaginase, Cyclophosphamide, 6-MP, Doxorubicin	No	No	16.7
UPN005	M	B-ALL	4.3	Vincristine, Daunorubicin, MTX, ARA-C, Asparaginase, Cyclophosphamide, 6-MP, Doxorubicin	No	No	5.1
UPN006	F	B-ALL	3.4	Vincristine, Daunorubicin, MTX, ARA-C, Asparaginase, Cyclophosphamide, 6-MP	No	No	3.6
UPN007	F	B-ALL	8.3	Vincristine, Daunorubicin, MTX, ARA-C, Asparaginase, Cyclophosphamide, 6-MP, Doxorubicin	No	No	9.4
UPN008	F	Osteosarcoma *Euramos1*	13.5	Doxorubicin, Cisplatin, MTX<br>If randomized/poor response: + Ifosfamide, Etoposide	NA	NA	14.9
UPN009	M	Non low-grade astriocytoma *SIOP LGG2004*	14.1	None	No	Yes	15.4
UPN010	M	Ewing *Ewing2008R3*	3.9	Ifosfamide, Doxorubicin, Actinomycin D, Vincristine, Cyclophosphamide, Etoposide	No	Yes	5.7
UPN011	F	Neuro-ganglioblastoma *NBL2009MRG*	3.3	Cisplatin, Etoposide, Vindesine, Vincristine, Dacarbazine, Ifosfamide, Doxorubicin, low dose Cyclophosphamide, Retinoic acid	No	Yes	5.9
UPN012	M	Neuroblastoma	4.6	Cisplatin or carboplatin, etoposide, Vindesine, Dacarbazine, Doxorubicin, Ifosfamide, Vincristine, Busulfan, Melfalan.	No	Yes	7.4
UPN013	M	b-thalassemia *NA*	NA	Treosulfan, Fludarabine, Thiotepa, ATG, Alemtuzumab	Yes, 2x	NA	5.3
UPN014	M	ALL *ALL11-MRG*	6.0	Vincristine, Daunorubicin, Asparaginase, Cyclophosphamide, ARA-C, 6-MP, MTX, Doxorubicin	No	No	7.1
UPN015	F	Lymphoma *NA*	NA	NA	NA	NA	15.6
UPN016	F	NHL *ALL VII*	NA	Daunorubicin, 6-TG, Vindesine, 6-MP, Asparaginase, Cyclophosphamide, Vincristine, Doxorubicin, Teniposide, MTX, ARA-C, Ifosfamide	No	No	11.2
UPN017	M	AML *ANLL92*	5.2	Doxorubicin, Cyclophosphamide, Idarubicin, ARA-C, Vincristine, Mitoxantrone, Etoposide, 6-TG	No	No	15.1
UPN018	M	pre-B ALL *ALL8-MRG*	4.6	Vincristine, Daunorubicin, Asparaginase, ARA-C, MTX, 6-MP, Doxorubicin, Cyclophosphamide, 6-TG	No	No	7.8
UPN019	M	AML *ANLL94*	7.1	ARA-C, Idarubicin, Etoposide, Mitoxantrone, 6-TG, Vincristine, Doxorubicin, Cyclophosphamide<br>Conditioning: + Busulfan	Yes	TBI	10.9
UPN020	M	ALL *NA*	8.2	NA	NA	NA	14.0
UPN022	F	Ewing sarcoma *NA*	NA	NA	NA	NA	9.1
UPN023	F	T-ALL *ALL10-MRG*	9.6	Vincristine, Daunorubicin, Asparaginase, Cyclophosphamide, 6-MP, ARA-C, Leukovorin, MTX, Doxorubicin	No	No	12.2
UPN024	M	T-LBL *Euro-LB02 III/IV*	8.4	Vincristine, Daunorubicin, MTX, Asparaginase, 6-MP, ARA-C, Cyclophosphamide, Doxorubicin, 6-TG,	No	No	10.0
UPN034	M	Osteosarcoma *Euramos1*	15.7	Doxorubicin, Cisplatin, MTX	No	No	15.9

*NOTE 1: all chemotherapy has been retrieved from clinical data or extracted to our best knowledge from stated protocols, this can vary per individual patient. NOTE 2: all IBFM-patient samples are collected from Germany, except for IBFM67, this sample was collected from Austria. [1]According to treatment protocol. [2]Proton therapy. [3] HLH diagnosis one year after JIA; 6-MP = Mercaptopurine; 6-TG = thioguanine; ALL = acute lymphoblastic leukaemia; alloSCT = allogenic stem cell transplantation; AML = acute myeloid leukaemia; ARA-C = cytarabin; BuFluClo = Busulfan, Fludarabin, Clofarabin; Dx1 = first diagnosis; F = female; HLH = Hemophagocytic Lymphohistiocytosis; HR = high risk; JIA = Juvenile Idiopathic Arthritis; M = male; MRG = medium risk group; MTX = methotrexate; MUD = matched-unrelated-donor; NA = not available; NF1 = neurofibromatosis type 1; NHL = non-Hodgkin lymphoma; RT = radiotherapy; SCT = stem cell transplantation; St = stage; TBI = total body irradiation; t-MN = therapy-related myeloid neoplasm; y = years.
