# Supplementary material for: Selective pressures of platinum compounds shape the evolution of therapy-related myeloid neoplasms
Source: Nat Commun. 2024 Jul 17;15:6025. doi: 10.1038/s41467-024-50384-z (PMC11255340; doi:10.1038/s41467-024-50384-z)
Supplement: Supplementary file 3 — Description of Additional Supplementary files [file 41467_2024_50384_MOESM3_ESM.pdf]

Description of Additional Supplementary files

Supplementary Data 1: Drivers, single base substitutions and mutational signatures per patient. Pe r tMN patient, the driving mutations and fusions, total numbers of single base substitutions (SBS), i nsertions and deletions (indels), double base substitutions (DBS) and the contribution of each muta tional signature to the SBS is depicted.
(separate file)

Supplementary Data 2: Number and type of cells sequenced for each phylogenetic tree. Bulk popul ations include t-MN blasts and mesenchymal stromal cells (MSC) or B-cells. (separate file)

Supplementary Data 3: Whole genome sequencing (WGS) metrics of every sample.
Supplementary Data 4: Per patient clinical trial and treatment protocol information
