# Supplementary material for: Selective pressures of platinum compounds shape the evolution of therapy-related myeloid neoplasms
Source: Nat Commun. 2024 Jul 17;15:6025. doi: 10.1038/s41467-024-50384-z (PMC11255340; doi:10.1038/s41467-024-50384-z)
Supplement: Supplementary file 8 — Reporting Summary [file 41467_2024_50384_MOESM8_ESM.pdf]

nature portfolio

Corresponding author(s): R. van Boxtel

Last updated by author(s): Apr 11, 2024

Reporting Summary

Nature Portfolio wishes to improve the reproducibility of the work that we publish. This form provides structure for consistency and transparency in reporting. For further information on Nature Portfolio policies, see our Editorial Policies and the Editorial Policy Checklist.

Statistics

For all statistical analyses, confirm that the following items are present in the figure legend, table legend, main text, or Methods section.

n/a	Confirmed	
☐	☒	The exact sample size (*n*) for each experimental group/condition, given as a discrete number and unit of measurement
☐	☒	A statement on whether measurements were taken from distinct samples or whether the same sample was measured repeatedly
☐	☒	The statistical test(s) used AND whether they are one- or two-sided<br>*Only common tests should be described solely by name; describe more complex techniques in the Methods section.*
☐	☒	A description of all covariates tested
☐	☒	A description of any assumptions or corrections, such as tests of normality and adjustment for multiple comparisons
☐	☒	A full description of the statistical parameters including central tendency (e.g. means) or other basic estimates (e.g. regression coefficient) AND variation (e.g. standard deviation) or associated estimates of uncertainty (e.g. confidence intervals)
☐	☒	For null hypothesis testing, the test statistic (e.g. *F*, *t*, *r*) with confidence intervals, effect sizes, degrees of freedom and *P* value noted<br>*Give P values as exact values whenever suitable.*
☐	☒	For Bayesian analysis, information on the choice of priors and Markov chain Monte Carlo settings
☐	☒	For hierarchical and complex designs, identification of the appropriate level for tests and full reporting of outcomes
☒	☐	Estimates of effect sizes (e.g. Cohen's *d*, Pearson's *r*), indicating how they were calculated

Our web collection on statistics for biologists contains articles on many of the points above.

Software and code

Policy information about availability of computer code

Data collection	Sony SH800s cell sorter software (v2.1.6), CytExpert Data (v2.4.0.28)
Data analysis	WGS data was processed with the NF-IAP pipeline v1.3.0 (https://github.com/UMCUGenetics/NF-IAP), the SMuRF pipeline v3.0.0 (https://github.com/ToolsVanBox/SMuRF) and the PTATO pipeline v1.3.3. (https://github.com/ToolsVanBox/PTATO). Code for all downstream analyses (in bash and R) is available upon request. Here, mainly the MutationalPatterns R package v3.6.0 was used. The following R packages were used for analysis of MV4-11 and UCB experiments: ggplot2 (v3.3.6), DRC (v.3.0-1), DRC (v.3.0-1), ggpubr (v.0.6.0), stats v4.2.2, v.0.7.2. Flow cytometry data of MV4-11 experiments were analysed using FlowJo(TM) (v10.8.1).

For manuscripts utilizing custom algorithms or software that are central to the research but not yet described in published literature, software must be made available to editors and reviewers. We strongly encourage code deposition in a community repository (e.g. GitHub). See the Nature Portfolio guidelines for submitting code & software for further information.

Data

Policy information about availability of data

All manuscripts must include a data availability statement. This statement should provide the following information, where applicable:

- Accession codes, unique identifiers, or web links for publicly available datasets
- A description of any restrictions on data availability
- For clinical datasets or third party data, please ensure that the statement adheres to our policy

The whole genome sequencing (WGS) data generated in this study have been deposited in the European Genome-phenome Archive (EGA) under accession code EGAS00001005141 [https://ega-archive.org/studies/EGAS00001005141]. The raw WGS data are available under restricted access due to privacy laws, access can be obtained via the Princess Máxima Data Access Committee [https://ega-archive.org/dacs/EGAC00001001864]. The processed WGS and other data needed to generate the figures have been deposited in the Mendeley Database [https://data.mendeley.com/datasets/9d7mhxzt9g/1]. The raw mutation data of the cohort of solid tumor metastases we obtained are available under restricted access due to privacy laws. Access can be obtained via the Hartwig Medical Foundation [https://www.hartwigmedicalfoundation.nl/en/data/data-access-request/].

Research involving human participants, their data, or biological material

Policy information about studies with human participants or human data. See also policy information about sex, gender (identity/presentation), and sexual orientation and race, ethnicity and racism.

Reporting on sex and gender	In this manuscript, the term sex has been stated for all included patients. This information is based on clinical data from either the Princess Maxima Center for pediatric oncology or the German Society of Pediatric Oncology and Hematology (GPOH). If these data were missing, these data were acquired from whole genome sequencing (WGS) results.
Reporting on race, ethnicity, or other socially relevant groupings	In this manuscript, there is no reporting on race, ethnicity or other socially relevant groupings.
Population characteristics	Patients with a clinical diagnosis of therapy-related myeloid neoplasm (t-MN) were included from the Princess Maxima Center. From Germany, patients with a clinical diagnosis of therapy-related AML (t-AML) were included. All included patients were below 20 years of age at the time of t-MN diagnosis. There has been no selection for genotypic information and treatment categories.
Recruitment	Pediatric cancer patients from the Netherlands were treated at the Princess Máxima Center for Pediatric Oncology, and recruited via the Princess Máxima Center biobank procedure. In addition, patients from Germany were collected via the national BFM studygroup following a a collaboration with the International Berlin-Frankfurt-Munster AML Study Group (I-BFM AML SG).
Ethics oversight	Patient samples were collected via the biobank of the Princess Máxima Center for Pediatric oncology and via a collaboration with the I-BFM AML SG from the German AML-BFM study group in accordance with the Declaration of Helsinki. Informed consents were obtained from all participants. For the Dutch samples, ethical approval was granted in the past for treatment according to the respective clinical trials the patients were treated in. These study protocols were approved by the involved ethics committee of the center the patient was treated at. Samples were collected and banked at the Dutch Childhood Oncology Group (DCOG), after informed consent for study participation was obtained. The DCOG transferred all clinical data and biological specimens with the merge of all pediatric oncology centers in the new facility (the Máxima) in 2018. In the Netherlands when re-using banked samples for wet-lab research that were originally stored with informed consent for additional research, no additional ethics committee approval is required, instead the Institutional Review Board of the Máxima approved this study under proposal PMCLAB2020.151.Umbilical cord blood was obtained from the Wilhelmina Children's Hospital after approval by the Biobank Committee of the University Medical Center Utrecht (protocol number 15-341).

Note that full information on the approval of the study protocol must also be provided in the manuscript.

Field-specific reporting

Please select the one below that is the best fit for your research. If you are not sure, read the appropriate sections before making your selection.

☒ Life sciences ☐ Behavioural & social sciences ☐ Ecological, evolutionary & environmental sciences

For a reference copy of the document with all sections, see nature.com/documents/nr-reporting-summary-flat.pdf

Life sciences study design

All studies must disclose on these points even when the disclosure is negative.

Sample size	Patient samples: No sample size calculation was performed. We collected and processed all available bone marrow and peripheral blood material of t-MN patients at time of t-MN diagnosis. The number of cells that was sequenced per patient was dependent on the availability and quality of the material upon work-up.<br>MV4-11 and UCB experiments: the number of seeded cells per condition were determined based on the plating densities of the material

	during standard culture (as described in methods). The experiments were performed in triplicates as is standard in experimental biology, which resulted in the use of three independent biological donors for the UCB experiments (no sample size calculation was performed), and the use of three clonal cultures per genotype for the MV4-11 experiments.
Data exclusions	No data were excluded from analyses.
Replication	A similar research set-up was performed in 44 patients. For the samples in which we performed phylogenetic lineage tracing, we performed the same analysis in at least 2 patients per subset (TP53+/+ N=5, and TP53+/- N=2). From these patients, we sequenced at least 6 single cells. For the MV4-11 and UCB experiments, each experiment was performed three times independently. Each attempt at replication was successful and incorporated into the manuscript.
Randomization	No randomization was performed for patients. For WGS of HSPCs after clonal expansion, the cells that showed clonal outgrowth were collected for WGS. For single-sell WGS using primary template-directed amplification (PTA), the cells were selected based on FACS data (viability, gating). Controlling of covariates was not applicable as the cohort was to small to do so.
Blinding	As all samples were analyzed using bioinformatic pipelines, no blinding was possible.

Reporting for specific materials, systems and methods

We require information from authors about some types of materials, experimental systems and methods used in many studies. Here, indicate whether each material, system or method listed is relevant to your study. If you are not sure if a list item applies to your research, read the appropriate section before selecting a response.

Materials & experimental systems

n/a	Involved in the study
☐	☒ Antibodies
☐	☒ Eukaryotic cell lines
☒	☐ Palaeontology and archaeology
☒	☐ Animals and other organisms
☒	☐ Clinical data
☒	☐ Dual use research of concern
☒	☐ Plants

Methods

n/a	Involved in the study
☒	☐ ChIP-seq
☐	☒ Flow cytometry
☒	☐ MRI-based neuroimaging

Antibodies

Antibodies used	CD34-BV421 (Biolegend clone 561, Catalog number:343610)<br>lineage (CD3/CD14/CD19/CD20/CD56)-FITC (Biolegend, clones UCHT1, HCD14, HIB19, 2H7, HCD56, Catalog number: 348801)<br>CD38-PE (BioLegend, clone HIT2, Catalog number: 303506)<br>CD90-APC (BioLegend, clone 5E10, Catalog number: 328114)<br>CD45RA-PerCP/Cy5.5 (Biolegend, clone HI100, Catalog number: 304028)<br>CD33-PE/Cy7 (BioLegend, clone WM53, Catalog number: 303433)<br>CD49f-PE/Cy7 (Biolegend, clone GoH3, Catalog number: 313621)<br>CD16-FITC (Biolegend, clone 3G8, Catalog number: 302006)<br>CD11c-FITC (Biolegend, clone 3.9, Catalog number: 301604)<br>CD123-Pe/Cy7 (Biolegend, clone 6H6, Catalog number: 306009)<br>CD13-PerCP/Cy5.5 (Biosciences, clone WM15, Catalog number: 561361)<br>CD14-APC (BioLegend, clone HCD14, Catalog number: 325607)<br>CD3-PE/Cy7 (BioLegend, clone SK7, Catalog number: 344815)<br>CD4-PerCP/Cy5.5 (BioLegend, clone OKT, Catalog number: 317427)<br>CD20-BV421 (BioLegend, clone 2H7, Catalog number: 302329)<br>CD33-APC (Biolegend, clone WM53, Catalog number: 303407)<br>CD34-APC (BioLegend clone 561, Catalog number: 343607).
Validation	The above antibodies have been validated for the purpose of flow cytometry applications by the supplier.<br><br>CD34-BV421: Validation statement Biolegend: FC - Quality tested. References: Rosendahl Huber A, et al. 2022. STAR Protoc. 3:101361. Brandsma AM, et al. 2021. Blood Cancer Discov. 2:484. Yuzuriha A, et al. 2021. Methods Mol Biol. 2454:411. de Kanter JK, et al. 2021. Cell Stem Cell. 28:1726. Vasu S, et al. 2016. Blood. 127: 2879 - 2889. Osorio FG et al. 2018. Cell reports. 25(9):2308-2316 . and others.<br>lineage (CD3/CD14/CD19/CD20/CD56)-FITC: Validation statement Biolegend: FC - Quality tested. References: Shafiei–Jahani P, et al. 2020. Nat Commun. 3.734722222. Doherty T, et al. 2012. Am J Physiol Lung Cell Mol Physiol. 303:577. Laustsen A, et al. 2021. Elife. 10:. Lal A, et al. 2021. Nat Commun. 1507:12. Hurrell BP, et al. 2019. Cell Rep. 29:4509. Imai Y, et al. 2021. JID Innov. 1:100003. Delaney C, et al. 2021. EMBO Mol Med. 13:e12889. and others.<br>CD38-PE: Validation statement Biolegend: FC - Quality tested. References: Kishimoto T, et al. Eds. 1997. Leucocyte Typing VI. Garland Publishing Inc. London. Dieu M. 1998. J. Exp. Med. 188:373. Esser M, et al. 2001. J. Virol. 75:6173. Jeannin P, et al. 1999. J. Immunol. 162:2044. Yoshino N, et al. 2000. Exp. Anim. (Tokyo) 49:97. (FC) and others.<br>CD90-APC: Validation statement Biolegend: FC - Quality tested. References: Gómez-Aristizábal A, et al. 2016. PLoS One. 11: 0147868. Veraitch O, et al. 2017. Sci Rep. 7:42777. Melzer C, et al. 2020. Int J Mol Sci. :21. Rosendahl Huber A, et al. 2022. STAR Protoc.

3:101361. Brandsma AM, et al. 2021. Blood Cancer Discov. 2:484.Pustlauk W, et al. 2020. Sci Rep. 10:5951. and others
CD45RA-PerCP/Cy5.5: Validation statement Biolegend: FC - Quality tested. References: Knapp W, et al. 1989. Leucocyte Typing IV. Oxford University Press. New York. Esser M, et al. 2001. J. Virol. 75:6173. Yamada T, et al. 2002. J. Biol. Chem. 277:28830. Nagano M, et al. 2007. Blood 110:151. Jiang Q, et al. 2008. Blood 112:2858. Lee J, et al. 2015. J Exp Med. 212:385. and others
CD33-PE/Cy7: Validation statement Biolegend: FC - Quality tested. References: Brandsma AM, et al. 2021. Blood Cancer Discov. 2:484. Hasaart KAL, et al. 2020. Sci Rep. 10:12991. Matsuo S, et al. 2021. PLoS One. e0247595:16.
CD49f-PE/Cy7: Validation statement Biolegend: FC - Quality tested. References: Cayre S, et al. 2020. Development. 147:00:00. Ludwik KA, et al. 2020. Cell Reports. 32(3):107931. Rosendahl Huber A, et al. 2022. STAR Protoc. 3:101361. Larsen SB, et al. 2021. Cell Stem Cell. 28:1758. de Kanter JK, et al. 2021. Cell Stem Cell. 28:1726. Romagnoli M, et al. 2020. Development. 147:. Xiao X, et al. 2019. Cell Discov. 5:2. and others
CD16-FITC: Validation statement Biolegend: FC - Quality tested. References: Knapp W, et al. Eds. 1989. Leucocyte Typing IV. Oxford University Press. New York. Timmerman KL, et al. 2008. J. Leukoc. Biol. 84:1271. Yoshino N, et al. 2000. Exp. Anim. (Tokyo) 49:97. Brainard DM, et al. 2009. J. Virol. 83:7305. and others
CD11c-FITC: Validation statement Biolegend: FC - Quality tested. References: Schlossman S, et al. Eds. 1995. Leucocyte Typing V. Oxford University Press. New York. Ottonello L, et al. 1999. Blood 93:3505. Sadhu C, et al. 2007. J. Leukoc. Biol. doi:10.1189/jlb.1106680. Yoshino N, et al. 2000. Exp. Anim. (Tokyo) 49:97. Sadhu C, et al. 2008. J. Immunoass. Immunoch. 29:42. and others
CD123-Pe/Cy7: Validation statement Biolegend: FC - Quality tested. References: Montes de Oca M, et al. 2016. Cell Rep. 17:399-412. Han L, et al. 2012. PLoS One. 4:e7989. Wieland S, et al. 2014. J Virol. 88:752. Yamauchi T et al. 2018. Cancer cell. 33(3):386-4. Hagan T, et al. 2020. Cell. 178(6):1313-1328.e13. Royle C, et al. 2014. J Immunol. 193:3538. de Boer B et al. 2018. Cancer cell. 34(4):674-689. Schwartz JA, et al. 2018. J Virol. 92:19. and others
CD13-PerCP/Cy5.5: Validation statement Biosciences: Flow cytometry (Routinely Tested). References: Barclay NA ed. Academic Press; 1997. Favaloro EJ, Br J Haematol. 1988; 69(2):163-171. Yang P, Cell Mol Immunol. 2020; 17(5):555-557. and others
CD14-APC: Validation statement Biolegend: FC - Quality tested. References: Colonna L, et al. 2016. Clin Immunol. 163:84-90. Guo X, et al. 2016. Mol Ther Methods Clin Dev. 3:15054. Xun X, et al. 2021. Am J Transl Res. 13:4360. Hearnden R, et al. 2021. STAR Protocols. 2(2):100422. Wang E, et al. 2021. Cell Stem Cell. 28(4):718-731.e6. and others
CD3-PE/Cy7: Validation statement Biolegend: FC - Quality tested. References: Alfonso-Dunn R, et al. 2022. Front Immunol. 13:926318. Liu R, et al. 2022. Aging Dis. 13:1576. Nakano M, et al. 2021. Front Immunol. 12:713225.Stras SF, et al. 2020. Developmental Cell. 51(3):357-373.e5. Nathan A, et al. 2021. Cell. 184(17):4401-4413.e10. Pascual–García M, et al. 2019. Nat Commun. 10:2416. and others.
CD4-PerCP/Cy5.5: Validation statement Biolegend: FC - Quality tested. References: Charpentier JC, et al. 2020. Nat Commun. 11:180. Mo Y, et al. 2022. Front Immunol. 12:799896. Chan JA, et al. 2022. Nat Commun. 13:4159. Qian Y, et al. 2021. Cell Reports. 36(8):109602. Kongsbak M, et al. 2014. PLoS One. 9:96695. Weymar GHJ, et al. 2022. Cell Rep. 40:111311. and others.
CD20-BV421: Validation statement Biolegend: FC - Quality tested. References: Weber MG, et al. 2021. JCI Insight. 6:. Du J, et al. 2014. Cancer Immunol Res. 2:878. Schanin J, et al. 2022. Commun Biol. 5:1226. Vierboom MPM, et al. 2020. NPJ Vaccines. 5:39. Wang J, et al. 2020. Cell. 183(7):1867-1883.e26. Dijkman K, et al. 2021. Cell Reports Medicine. 2(1):100187. and others
CD33-APC: Validation statement Biolegend: FC - Quality tested. References: Harder L, et al. 2013. J Exp Med. 210:2289. Paris J et al. 2019. Cell Stem Cell. 25(1):137-148 . Frame JM, et al. 2020. Developmental Cell. 55(2):133-149.e6. Grenga I, et al. 2016. Clin Transl Immunology. 0.265972222. Arvindam US, et al. 2021. Leukemia. 35:1586. Burel JG, et al. 2019. Elife. 8. and others
CD34-APC: Validation statement Biolegend: FC - Quality tested. References: Mendt M, et al. 2018. JCI Insight. 9:1523. Park J, et al. 2021. Exp Ther Med. 22:808. Liu Y, et al. 2022. Signal Transduct Target Ther. 7:347. Lee JK, et al. 2020. Sci Rep. 2.567361111. Guzzi N et al. 2018. Cell. 173(5):1204-1216 . Riether C, et al. 2021. Cell Reports. 34(4):108663. and others

Eukaryotic cell lines

Policy information about cell lines and Sex and Gender in Research

Cell line source(s)	The MV4-11bulk and MV4-11R248W cells were kindly provided by the Frank van Leeuwen and Willem Cox (Princess Máxima Center for pediatric oncology, Utrecht, The Netherlands). Both lines are derived from pediatric acute monocytic leukemia of a 10 year old male patient. Of these, clonal cultures were derived through single-cell sorting and clonal expansion (this manuscript).
Authentication	The cell line identity was confirmed by STR profiling and comparison to the DSMZ CellDive database when the cell line was received. The presence of the TP53 mutation was confirmed using Sanger Sequencing.
Mycoplasma contamination	All cell lines tested negative for mycoplasma every six weeks.
Commonly misidentified lines (See ICLAC register)	No commonly misidentified lines were used in this study.

Flow Cytometry

Plots

Confirm that:

☒ The axis labels state the marker and fluorochrome used (e.g. CD4-FITC).

☒ The axis scales are clearly visible. Include numbers along axes only for bottom left plot of group (a 'group' is an analysis of identical markers).

☒ All plots are contour plots with outliers or pseudocolor plots.

☒ A numerical value for number of cells or percentage (with statistics) is provided.

Methodology

Sample preparation	Cells were stained using the HSPC antibody staining mix including specific leukemic blast markers, or the mature mix for T0- and B-cells (methods) for 60 minutes on ice. Cells were washed using FACS buffer (0.05% BSA + 1 mM EDTA in PBS) before sorting.<br>For proliferation and viability assays, cells were stained using CellTrace(TM) Far Red (Invitrogen) and DAPI in PBS.
Instrument	Single cells and bulk blasts and mature populations were sorted using a Sony SH800s cell sorter.<br>Proliferation and viablitity for cell line experiments was assessed using the CytoFLEX S (Beckman Coulter).
Software	Sony SH800s system software was used to inspect gating and sort cells, FlowJo (v10.8.1) was used to generate a gating strategy figure. For the viability and proliferation assays, gated frequencies were expored from FlowJo and analyzed in R packages stats (v4.2.2), DRC (v.3.0-1), and rstatic (v.0.7.2).
Cell population abundance	For patients' HSPCs, single cells were sorted, so no post-sort analysis could be performed. For t-MN blasts, variant allele frequency (VAF) of sequenced population was determined to verify that this population was clonal. For MV4-11 cell lines, the frequency of the TP53 R248W variant was assessed by Sanger Sequencing and SNV discovery (Indigo Gear Genomics).
Gating strategy	A example figure is available in Extended Data figure for the cell line sorting. The HSPC isolation was published multiple times before (e.g., https://doi.org/10.1158/2159-8290.CD-22-0120).<br>For the HSPCs the following gating strategy was used: First gate: cells -> Second gate: Singlets -> Third: Lineage negative -> Fourth: CD34+ cells -> Fifth: CD38- CD45RA-<br>Blasts were gated based on diagnostic immunophenotyping data if available, in most cases blasts were CD33, CD38 and/or CD34 positive.<br>B- and T-cells were gated on: first gate: cells -> second gate: singlets -> third gate: negativity for blast markers (CD33/CD34) -> fourth gate either CD3+CD4+ (T-cells) or CD3-CD20+ (B-cells).<br><br>For cell-line experiments, the following gating strategy was used to identify the live cell fraction: first gate: Cells -> second gate: Singlets -> third: DAPI negative.

☒ Tick this box to confirm that a figure exemplifying the gating strategy is provided in the Supplementary Information.
